# Artificial intelligence in football talent identification: a systematic review

**DOI:** 10.3389/fspor.2026.1770231

**Published:** 2026-08-20

**Authors:** Rui Zhou, Jorge Arede, Xinbi Zhang, Xiaocen Hao, Yingzhe Song, Zhimo Jiang, Bingnan Gong, Nuno Leite

**Affiliations:** 1 Capital University of Physical Education and Sports, Beijing, China; 2 Instituto Politecnico de Viseu, Viseu, Portugal; 3 Universidade de Tras-os-Montes e Alto Douro, Vila Real, Portugal; 4 Centro de Investigacao em Desporto Saude e Desenvolvimento Humano, Vila Real, Portugal; 5 Universidade do Porto, Porto, Portugal

**Keywords:** artificial intelligence, football talent identification, machine learning, player performance analysis, predictive modelling

## Abstract

**Background:**

The rapid development of artificial intelligence (AI) in sports has driven a shift in football talent identification from traditional experience-based judgment to data-driven decision-making. However, existing evidence on the application of AI in talent identification is dispersed, and a systematic synthesis of its application methods and effectiveness across different stages of the identification process is lacking.

**Objective:**

To systematically review the application of AI in football talent identification, compare sample characteristics, model types, data sources, and application contexts across different AI techniques, and summarize their advantages, limitations, and future directions.

**Methods:**

This systematic review followed PRISMA guidelines and was registered with PROSPERO. Searches were conducted in Web of Science, Scopus, SportDiscus, and PubMed to identify eligible studies, which were assessed for methodological quality. Information regarding sample characteristics, AI technique type, input features, model performance, and application context was extracted and synthesized.

**Results:**

A total of 20 studies were included, covering youth and professional male players. Traditional machine learning models were the most frequently used, whereas deep learning and hybrid models were less common. AI demonstrated potential in three main areas: developmental potential identification, performance and role identification, and player value and recruitment decision-making. Although most studies adopted multidimensional feature sets, limitations remain regarding data quality, model interpretability, methodological rigor, and external validity.

**Conclusion:**

AI has shown substantial value in football talent identification, but current research is still transitioning from methodological exploration to systematic application. Future work should focus on developing more representative datasets, promoting cross-institutional data sharing, enhancing model transparency and interpretability, and validating AI models in real-world football contexts to ensure their safe, reliable, and effective application in talent identification practice.

**Systematic Review Registration:**

https://www.crd.york.ac.uk/PROSPERO/view/CRD420251196817, PROSPERO: CRD420251196817.

## 1 Introduction

Talent identification is one of the core topics in sports science research, particularly in the context of competitive sports. Athletic talent is often defined as an individual's exceptional potential in a specific sport (1). Individuals with such potential are considered more likely to excel in training and competition (2). Based on this perspective, practitioners tend to prioritize identifying these individuals and including them in elite athlete development systems (3). However, as researchers gain a deeper understanding of athlete development pathways, the notion of “innate talent” has faced increasing criticism. Critics argue that this view overlooks the critical influence of environmental, cultural, and social factors in the identification and development of talent (4, 5). Current research generally suggests that athletic talent should no longer be regarded as an innate, static trait but rather as a dynamic, multidimensional system of potential shaped by interactions among multiple factors (6–8). This emerging perspective highlights the adaptability and malleability of athletic talent, emphasizing that talent identification should be a long-term, dynamic evaluation process conducted across diverse environments (9). At the same time, this shift also prompts practitioners to re-examine existing talent identification systems.

Globally, sports organizations and clubs invest substantial resources in identifying and developing talent, especially in highly professionalized team sports such as football (10). For example, Category One football academies in England allocate annual budgets ranging from £2.3 million to £4.9 million for youth development (11). However, many academy players fail to progress into higher-level professional pathways during adulthood, not only wasting resources but also exposing the limitations of current talent identification systems (12). Traditional approaches often rely on single-dimensional physical or physiological measures, alongside subjective assessments by coaches and scouts (13). Although the “coach's eye” can, in some cases, effectively identify the future potential of young players, this approach often introduces biases due to its emphasis on short-term performance or biological maturation level, leading to the over-selection of early-maturing players (14–16). This not only reduces selection efficiency but also increases injury risks (17). Therefore, given the high investment and high attrition rates in contemporary football academies, improving the accuracy and validity of talent identification through systematic and scientific means has become a key issue for clubs and governing bodies (18).

In recent years, the rapid development of artificial intelligence (AI) has brought transformative changes to traditional practices in the sports domain (19). As an important branch of computer science, AI encompasses key technological fields such as machine learning (ML), deep learning (DL), and computer vision (CV), which have been widely applied to match prediction, action recognition, and performance analysis (20–22). The data-driven nature of AI also provides a new technical framework for football talent identification. Machine learning models such as Support Vector Machines (SVM), XGBoost, and Random Forests (RF) have demonstrated strong potential in player selection and positional analysis (23, 24). Deep learning models such as deep neural networks (DNNs) have also been used to predict player potential and developmental trajectories based on multidimensional features (e.g., match performance, technical statistics, and physiological data), offering new quantitative tools for selection decisions (25).

Similar to other fields, the practical implementation of AI in football talent identification still faces challenges such as data security, algorithmic bias, and insufficient model interpretability (26). Therefore, before further adopting and promoting AI technologies in talent identification and development, it is necessary to systematically summarize, compare, and critically analyze the current landscape. Although a small number of systematic reviews have examined the applications of AI in football, these studies have mainly focused on match performance analysis, tactical recognition, and injury prediction (27–29). In contrast, systematic investigations specifically addressing the applications, technical types, and limitations of AI in football talent identification and development remain extremely limited.

The introduction of AI technologies is reshaping the evaluative logic of youth development systems, shifting talent identification from experience-driven judgments to data-driven, objective decision-making. Without systematic reviews that synthesize and compare existing evidence, researchers and practitioners will find it difficult to rigorously assess the effectiveness, reliability, and potential risks of AI in talent development, thereby limiting its meaningful integration into football talent identification. Therefore, this study aims to systematically review and evaluate the current applications of AI in football talent identification and development, compare the characteristics of different algorithmic models, summarize their advantages and limitations, and propose future directions for research and practice.

## 2 Materials and methods

This systematic review followed the Preferred Reporting Items for Systematic Reviews and Meta-Analyses (PRISMA) guidelines (30, 31). The review protocol was prospectively registered in the International Prospective Register of Systematic Reviews (PROSPERO) (Registration ID: CRD420251196817).

### 2.1 Search strategy

A comprehensive literature search was conducted on June 15, 2025, across the following major electronic databases, including Web of Science, Scopus, SportDiscus, and PubMed, to identify studies examining the application of artificial intelligence in football talent identification. The search combined keywords and subject headings (including MeSH terms in PubMed) covering the following core concepts: (i) “football” or “soccer”; (ii) “artificial intelligence,” “machine learning,” “deep learning,” or “neural network”; and (iii) “talent identification,” “scouting,” “player selection,” or related terms concerning player prediction and evaluation (see Table 1). In addition, the reference lists of all included studies were manually screened to reduce the risk of missing relevant literature. The complete database-specific search strategies, including the exact search strings, search fields, filters, search dates, and number of records retrieved from each database, are provided in Supplementary Table 1. No publication-year restriction was applied at the search stage. Language and publication-type restrictions were applied during eligibility screening. Grey literature, including conference proceedings, preprints, and industry reports, was not systematically searched, as the review was restricted to peer-reviewed original research to enhance methodological consistency and quality control.

**Table 1:** Summary of databases searched and initial records retrieved.

Database	Search Query	Results
Web of Science	TS = (“artificial intelligence” OR “machine learning” OR “deep learning” OR “neural network”) AND TS = (“football” OR “soccer”) AND TS = (“talent” OR “talent identification” OR “scouting” OR “player selection” OR “player prediction” OR “transfer”)	249
PubMed	(“artificial intelligence”[Mesh] OR “machine learning”[Mesh] OR “neural networks, computer”[Mesh] OR “artificial intelligence”[tiab] OR “machine learning”[tiab] OR “deep learning”[tiab] OR neural network[tiab]) AND [“Soccer”[Mesh] OR football[tiab] OR soccer[tiab]] AND (talent[tiab] OR “talent identification”[tiab] OR scouting[tiab] OR “player selection”[tiab] OR player prediction[tiab] OR transfer[tiab])	109
SportDiscus	TX (“artificial intelligence” OR “machine learning” OR “deep learning” OR “neural network”) AND TX (“football” OR “soccer”) AND TX (“talent” OR “talent identification” OR “scouting” OR “player selection”)	155
Scopus	TITLE-ABS-KEY(“artificial intelligence” OR “machine learning” OR “deep learning” OR “neural network”) AND TITLE-ABS-KEY(“football” OR “soccer”) AND TITLE-ABS-KEY(“talent” OR “talent identification” OR “scouting” OR “player selection” OR “player prediction” OR “transfer”)	93

### 2.2 Eligibility criteria and study selection

**“**Talent” is a dynamic and multidimensional construct (32). Accordingly, talent identification should not be restricted solely to youth stages, as selection and evaluation processes also occur throughout an athlete's professional career, such as in player transfers, positional assessments, and long-term performance prediction (28, 33, 34). In the present review, “talent” was operationalised based on the outcome variables used in the included studies, rather than a single predefined definition. Specifically, football talent identification was understood as a broad decision-support process through which player qualities are identified, evaluated, profiled, or projected to inform selection, development, recruitment, and squad-building decisions. Under this definition, studies were considered relevant if they assessed player-related outcomes reflecting developmental potential, current performance, positional suitability, future performance, market value, transfer success, or recruitment value within a football context. These outcomes were treated as distinct decision-making problems within the broader player development and recruitment pathway, rather than as identical prediction tasks. This broad conceptualization of “talent” informed the inclusion criteria of the present review. Studies were included if they met the following criteria: (1) written in English; (2) published as original research in peer-reviewed journals; (3) reported data related to football players or teams; (4) involved competitive athletes, including professional, semi-professional, youth elite, national team, or collegiate players; and (5) explicitly described artificial intelligence technologies or algorithms applied to football talent identification. Studies were excluded if they: (1) focused on sports other than football; (2) were reviews, editorials, or non-peer-reviewed publications; or (3) introduced theoretical models without empirical validation.

All records retrieved from the database search and citation searching were imported into reference management software, and duplicates were removed before screening. Title/abstract screening and full-text eligibility assessment were performed independently by two reviewers (R.Z. and X.Z.) according to the predefined inclusion and exclusion criteria. Disagreements at either stage were resolved through discussion, and when consensus could not be reached, a third reviewer (Y.S.) adjudicated the decision. Screening was conducted independently and was not blinded to author or journal information. The study selection process was documented in a PRISMA 2020 flow diagram.

### 2.3 Data analysis

The following information was extracted from each included study: first author and year of publication, sample characteristics [following the methodology used in previous studies (35), we classified the sample based on soccer skill level], type of artificial intelligence technique used, input features or variables, software employed, performance metrics (such as accuracy, precision, recall, F1-score, or AUC), and application context. All extracted data were organized and synthesized in Excel spreadsheets.

### 2.4 Risk of bias assessment

Because the included studies primarily evaluated prediction-oriented artificial intelligence models, but differed in modelling aims, data structures, and analytical frameworks, risk of bias was assessed using an adapted domain-based approach informed by established prediction-model appraisal guidance, particularly PROBAST and its recent AI-oriented update (36, 37).

The assessment framework was structured around four core domains: participants/data sources, predictors, outcomes, and analysis. Each study was judged at the domain level as having low risk, high risk, or unclear risk of bias, and study-specific justifications were recorded accordingly.

In applying this framework to the present review, particular attention was given to issues especially relevant to AI-based football talent identification studies, including sample representativeness, authenticity of data sources, predictor definition and timing, outcome operationalisation, model overfitting, validation strategy, handling of class imbalance, potential feature leakage, and transparency of model reporting.

Two reviewers (R.Z. and X.Z.) independently performed the risk-of-bias assessment, and disagreements were resolved through discussion with a third reviewer (Y.S.).

The domain definitions applied in the present review are summarized in Table 2, and the study-level judgments are presented in Table 3. Detailed domain-level judgments and one-line justifications for each included study are provided in Supplementary Table 3.

**Table 2:** Adapted domain-based framework for risk-of-bias assessment of included studies.

Domain	Main considerations	Typical sources of high or unclear risk
Participants/data sources	Representativeness of the sample, authenticity of the data source, appropriateness of the study population for the modelling aim	Selective or highly restricted samples, small cohorts, single-player analyses, virtual/game-based datasets with limited real-world correspondence, unclear data provenance
Predictors	Clarity of predictor definition, timing of predictor availability, preprocessing and feature construction	Poorly defined predictors, unclear preprocessing, use of variables that may not be available at real decision time, possible feature leakage
Outcomes	Clarity and appropriateness of the target outcome, consistency with football talent identification aims	Vaguely defined outcomes (e.g., loosely defined “talent” or “quality”), unclear labelling procedures, mismatch between outcome and applied context
Analysis	Model development transparency, validation strategy, overfitting risk, class imbalance handling, completeness of performance reporting	Internal validation only, no external validation, limited reporting of tuning procedures, unclear handling of class imbalance, small-sample/high-dimensional modelling, incomplete performance metrics

The framework was adapted from prediction-model risk-of-bias principles and tailored to AI-based football talent identification studies in response to reviewer recommendations.

**Table 3:** Study-level risk-of-bias judgments for the included studies using the adapted domain-based framework.

Reference	Participants/data sources	Predictors	Outcomes	Analysis	Overall risk of bias	External validation	Key concern(s)
Developmental Potential Identification							
(Altmann et al., 2024) (24)	Low	Low	Low	Unclear	Unclear	Not reported	Single-academy cohort; internal validation only;
(Barron et al., 2018) (81)	Low	Low	Low	Unclear	Unclear	Not reported	Historical cohort; ANN opacity; no external validation reported
(Barron et al., 2020) (82)	Low	Low	Low	Unclear	Unclear	Not reported	Position-specific modelling; ANN opacity; no external validation reported
(Duncan et al., 2024) (43)	Unclear	Low	Unclear	High	High	Not reported	Small youth sample; very high accuracy; internal validation only
(Jauhiainen et al., 2019) (23)	Low	Low	Unclear	Unclear	Unclear	Not reported	Anomaly-detection design; elite label assumptions; no external validation reported
(Kelly et al., 2022) (44)	Unclear	Low	Unclear	High	High	Not reported	Small academy sample; exploratory design; performance metrics not clearly emphasised
(Sheng et al., 2021) (83)	High	Unclear	Unclear	High	High	Not reported	Single-team setting; system-development focus; limited generalisability
Performance & Role Identification							
(Dick et al., 2021) (56)	Low	Low	Unclear	Unclear	Unclear	Not reported	Indirect outcome; no external validation reported
(García-Aliaga et al., 2021) (55)	Low	Low	Unclear	Unclear	Unclear	Not reported	Provider-labelled positions; unsupervised structure; no external validation reported
(Khan et al., 2021) (84)	High	Unclear	High	High	High	Not reported	Single-player design; poor generalisability
(Jamil et al., 2021) (85)	Low	Low	Low	Unclear	Unclear	Not reported	Internal validation only; no external validation reported
(Merlin et al., 2024) (51)	Unclear	Low	Unclear	Unclear	Unclear	Not reported	Few matches; limited representativeness; no external validation reported
Player Value & Recruitment Identification							
(Arndt & Brefeld, 2016) (86)	Low	Low	Unclear	Unclear	Unclear	Not reported	Indirect outcome; sparse player-level data; no external validation
(Behravan & Razavi, 2021) (70)	High	Low	Low	High	High	Not reported	Virtual dataset; overfitting risk; no external validation
(Ćwiklinski et al., 2021) (33)	Low	Unclear	High	Unclear	High	Not reported	Custom transfer-success outcome; no external validation
(Inan & Cavas, 2021) (69)	Unclear	Low	Low	High	High	Not reported	Small position-specific sample; weak model performance
(Al-Asadi & Tasdemır, 2022) (67)	High	Low	Low	Unclear	High	Not reported	Virtual dataset; limited real-world generalisability
(Nouraie et al., 2023) (25)	High	Unclear	High	High	High	Not reported	Proof-of-concept design; non-standard outcome; no external validation
(van Arem et al., 2025) (68)	Unclear	Low	Unclear	Low	Unclear	Not reported	Selection bias risk; model-derived outcomes; no external validation
(Yigit et al., 2020) (71)	High	Low	Low	Unclear	High	Not reported	Virtual predictors; limited real-world representativeness

## 3 Results

### 3.1 Search results and study selection

**Figure 1:**
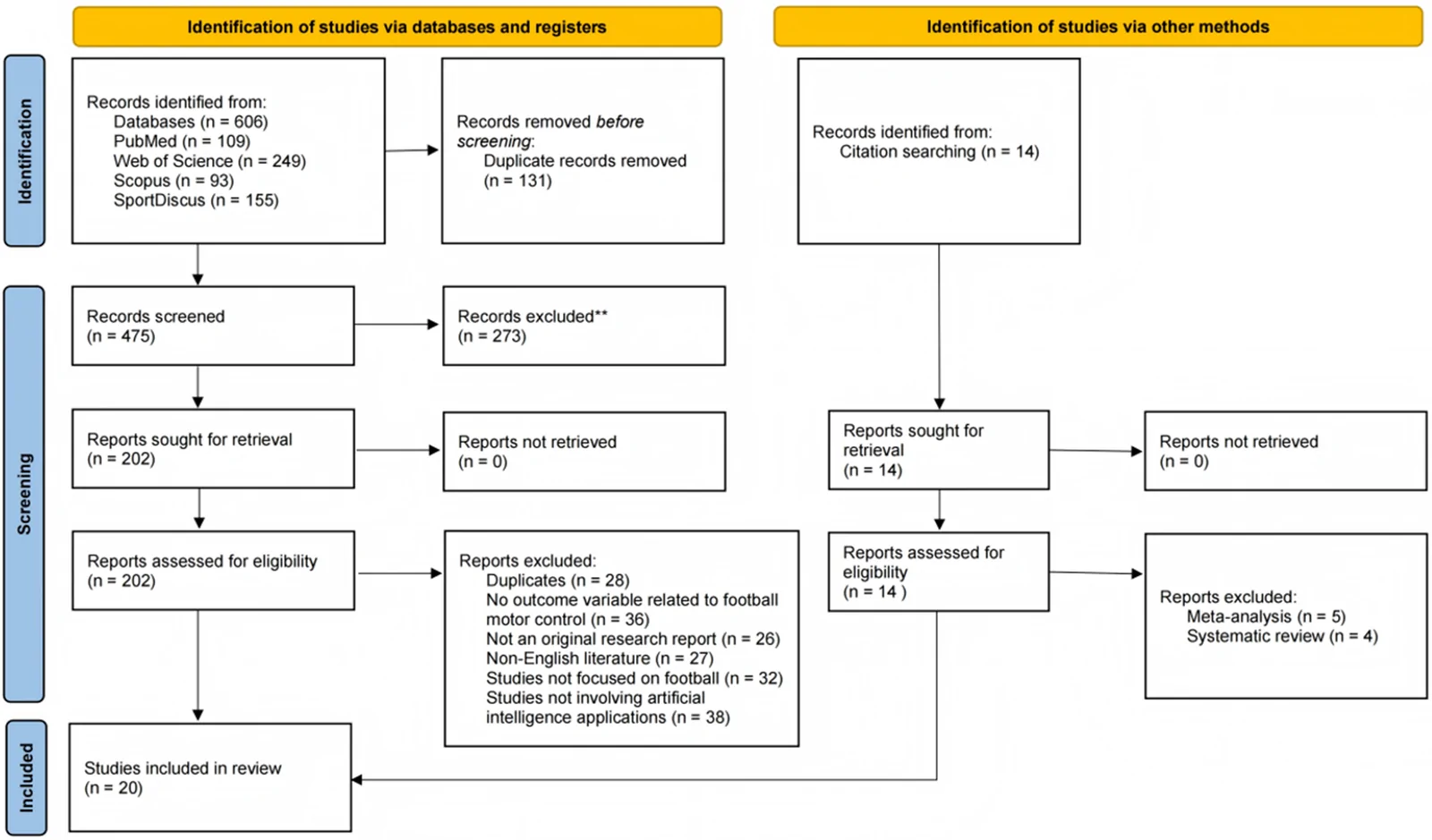
Flow chart for study inclusion and exclusion process based on PRISMA.

The database search identified 606 records (Web of Science, *n* = 249; PubMed, *n* = 109; SportDiscus, *n* = 155; and Scopus, *n* = 93). After removal of 131 duplicates, 475 records remained for title and abstract screening, of which 273 were excluded. As a result, 202 reports were sought for retrieval and assessed for eligibility, and 187 were excluded for the following reasons: duplicates (*n* = 28), no outcome related to talent identification, player evaluation, or recruitment decision-making (*n* = 36), not an original research report (*n* = 26), non-English literature (*n* = 27), not focused on football (*n* = 32), and not involving artificial intelligence applications (*n* = 38). In addition, 14 records were identified through citation searching. All 14 reports were assessed for eligibility, of which 9 were excluded (meta-analysis, *n* = 5; systematic review, *n* = 4). Ultimately, 20 studies were included in the review. The flowchart summarizing the selection process is presented in Figure 1.

### 3.2 Risk of bias assessment

Risk of bias was reassessed using the adapted domain-based framework described in Table 2, and the study-level judgments are presented in Table 3. A complete PROBAST-informed domain-level risk-of-bias table with study-specific justifications is provided in Supplementary Table 3. Across the included studies, the analysis domain showed the most frequent concerns. These were mainly related to limited reporting of model development procedures, insufficient information on tuning and preprocessing, reliance on internal validation only, and the absence of clearly reported external validation. In several studies, these issues increased the likelihood of optimistic performance estimates.

Additional concerns were identified in the participants/data sources domain. Although several studies used real-world football datasets, others relied on highly selective samples, single-team or single-player designs, or virtual datasets derived from football simulation platforms. These characteristics limited the representativeness of the study populations and reduced the generalisability of the reported findings.

In the predictors domain, risk was generally lower when variables were clearly defined and derived from structured testing, event, tracking, or player-profile data. However, some studies provided insufficient detail on feature construction or preprocessing procedures, resulting in unclear risk judgments. In the outcomes domain, studies were judged more favorably when the target outcome was explicitly defined, such as progression to a higher competitive level or market value prediction. By contrast, risk was more frequently judged as unclear or high when studies used indirect, provider-labelled, or non-standard outcomes related to performance quality, positional classification, or proof-of-concept system outputs.

Overall, only a limited number of studies were judged to have comparatively lower risk across most domains, whereas many studies presented at least one domain with high or unclear risk of bias. The most common recurring concerns were limited external validation, small or selective samples, the use of virtual datasets, and insufficient analytical transparency. These findings indicate that the current evidence base remains methodologically heterogeneous and should be interpreted with caution.

### 3.3 Characteristics of included studies

**Figure 2:**
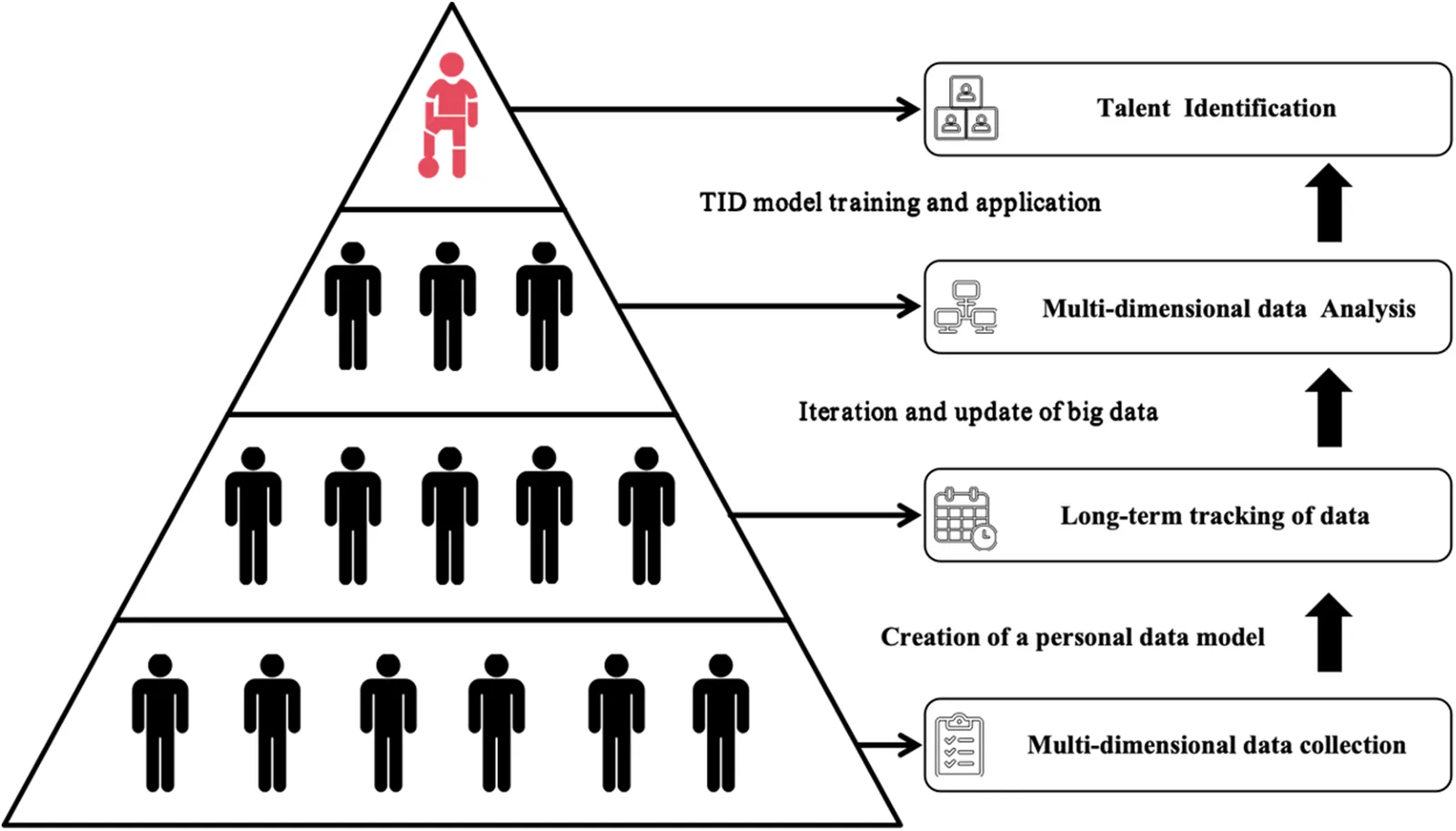
AI-based football talent identification model. This figure is based on a synthesis of the studies included in this review and is intended to summarize common processes and relationships reported in the literature, rather than to propose a new theoretical or practical framework.

A total of 20 studies were included in this review, and their main characteristics are presented in Table 4. The application process of artificial intelligence in talent identification is illustrated in Figure 2.

**Table 4:** Detailed characteristics of the included studies.

Author	Sample	Gender	Level	Input Features	AI Techniques	AI Task/Model Output	Software	Performance Metrics	Application
Developmental Potential Identification									
Altmann et al., 2024 (24)	409 youth players (U12–U19), seasons 2016–2023; (Germany)	Male	Highly Trained	16 multidimensional test indicators, including speed, skill, psychological measures, injury days, relative age, playing position, etc.	XGBoost*, RF, Lasso, Ridge	Classification: progression/selection outcome	Python	Accuracy=0.75 Precision = 0.84 Recall = 0.85 F1-Score = 0.84 AUC = 0.69	Predicting whether players would progress to higher age categories and identifying youth players with developmental potential.
Barron et al., 2018 (81)	966 outfield professional players, seasons 2008–2010; (England, UK)	Male	Elite	335 technical–tactical performance variables and 12 career-history variables.	ANN	Multiclass classification: future league status	Statistica	Prediction accuracy =61.5%–78.8%	Developing predictive models based on technical performance and career variables to identify players with the potential to progress to higher-level leagues.
Barron et al., 2020 (82)	966 outfield professional players, seasons 2008–2010; (England, UK)	Male	Elite	340 multidimensional indicators, including technical statistics, career data, and international competition participation, etc.	ANN	Position-specific multiclass classification: future league status	Statistica + Microsoft Visual Basic	Prediction accuracy = 72.7%–100%	Identifying key performance indicators across different playing positions and recognizing players with the potential to progress to higher-level leagues.
Duncan et al., 2024 (43)	162 youth players (U8–U14); (United Kingdom)	Male	Trained	11 multidimensional variables including anthropometrics, maturity, fitness, FMS, psychological perception, and coach ratings, etc.	RF*, LinR, Lasso, Ridge, BT	Classification: technical-skill performance level	Python	Accuracy = 98.6%	Identifying key factors that predict youth football technical performance and recognizing players with developmental potential.
Jauhiainen et al., 2019 (23)	951 youth players (U14); (Finland)	Male	Highly Trained	16 physical and technical indicators (speed, agility, technical skills, etc.) and 18 self-assessment questionnaire items (offense, defense, control, passing, 1v1, etc.).	One-Class SVM (RBF kernel)*, One-Class SVM (Linear kernel)	Anomaly detection: elite-potential outliers	Python	AUC=0.763	Using an anomaly detection framework to identify potential elite players from a large pool of typically developing youth players.
Kelly et al., 2022 (44)	98 youth players (U9–U16) and 18 youth players (U18); (England, UK)	Male	Highly Trained	53 multidimensional test variables, including technical–tactical, physical, psychological, and social factors, etc.	Lasso	Regression/classification: review rating or contract outcome	R	Not Specified	Identifying key multidisciplinary factors associated with youth players’ developmental potential.
Sheng et al., 2021 (83)	Training and match videos from an U20 men's team; (China)	Male	Highly Trained	Player and ball tracking data extracted from match videos, along with automatically generated technical–tactical statistical features.	BLS, RDBLS	Scoring/detection: ability score and potential-player support	-	Detection accuracy = 98.26%	Automated scoring, ability assessment, and identification of potential youth players.
Performance & Role Identification									
Dick et al., 2021 (56)	Tracking and event data from 54 professional league matches; (Germany)	Male	Elite	Feature set derived from tracking data and event data.	GRNN	Scoring/classification: action quality/possession value	-	AUC<0.64	comparing the quality of player actions, and identifying high-performing players.
Garcia-Aliaga et al., 2021 (55)	30,000 professional players, seasons 2012–2019; (Europe)	Male	Elite	52 technical–tactical performance features (classified into offensive, organisational, and defensive event categories).	t-SNE, UMAP, RIPPER	Dimensionality reduction/rule extraction: position and player profile	WEKA	Not Specified	Identifying and locating players with specific qualities to optimize team positional structure.
Khan et al., 2021 (84)	Lionel Messi's professional career data since 2005; (Spain)	Male	World Class	6 variables including Messi's participation, whether he scored, goals scored by other teammates, team total goals, opponent goals, and match result.	ABPNN	Classification: team-success dependence on key player	MATLAB	Accuracy = 94.96% Precision = 13.73% Recall = 86.27% Specificity = 95% F1-score = 0.904	Assessing whether a team is dependent on a specific player for winning, used to identify key players.
Jamil et al., 2021 (85)	353 professional goalkeepers, seasons 2013–2018; (Europe)	Male	Elite	73 GK technical performance indicators including passing, distribution, catching, shot-stopping, etc.	LR*, RFC, GBC	Binary classification: elite goalkeeper status	-	Accuracy = 0.671 F1-score = 0.664 AUC = 0.729	Differentiating goalkeepers who have competed in the UEFA Champions League from those who have not, to identify elite goalkeepers.
Merlin et al., 2024 (51)	2,522 passing events from 4 professional football matches; (Brazil)	Male	Elite	35 technical–tactical variables extracted from 2D positional data.	SVM*, RF, LR, KNN, LDA, NB, MLP, LSVM	Classification/ranking: pass difficulty and passing performance	Python	Balanced Accuracy = 70% F1-score = 0.71	Comparing players’ passing performances and identifying those with superior passing skills.
Player Value & Recruitment Identification									
Arndt & Brefeld, 2016 (86)	1,000 professional players, seasons 2009–2014; (Germany)	Male	Elite	match event data, player personal information, and team information.	SVR*, MT-RR, MT-SVR, RR, RFE, GRFE	Regression/forecasting: future player performance	-	Not Specified	Predicting player market value to support club decision-making in recruitment.
Behravan & Razavi, 2021 (70)	18,278 professional players (FIFA 20 dataset); (Game)	Male	Elite	55 player attributes, including offensive, defensive, physical characteristics, nationality, club, and skill-related metrics.	PSO-SVR* APSO-Clustering	Clustering/regression: player group and market value	MATLAB	MSE = 7.94 × 10^12^ RMSE = 2.81 × 10⁶ MAE = 7.11 × 10⁵ MAPE = 42.26 R^2^ = 0.74	Player classification and market value prediction for transfer valuation and recruitment decisions.
Ćwiklinski et al., 2021 (33)	3,482 professional players; (Europe)	Male	Elite	10 player and team variables including age, technical attributes, psychological traits, and team composite ratings.	RF*, Naive, Bayes, AdaBoost	Classification: transfer success/recruitment fit	-	Accuracy = 0.82 precision = 0.84 recall = 0.82 F1-score = 0.8	Predicting transfer success rate and identifying potential recruits who fit team requirements.
Inan & Cavas, 2021 (69)	236 professional midfielders, season 2018–2019); (Turkey)	Male	Elite	11 match performance variables including minutes played, goals, xG, assists, etc.	ANN	Regression: market value	MATLAB	R = –0.01994	Predicting players’ market value based on performance indicators to support recruitment and transfer decisions.
Al-Asadi & Tasdemir, 2022 (67)	17,980 professional players (FIFA 20 dataset); (Game)	Male	Elite	9 player rating indicators including age, height, potential, dribbling, shooting, position, and international reputation.	RF*, LinR, MT-LinR, DT	Regression: market value	Python	RMSE = 1.64, R^2^ = 0.95	Predicting player market value to support club transfer negotiations and target identification.
Nouraie et al., 2023 (25)	18,034 professional players, seasons 2016–2022; (Global)	Male	Elite	48 technical and physical variables including speed, shooting, passing, dribbling, height, weight, and preferred foot, etc.	DNN	Recommendation/matching: position suitability	-	Not Specified	Matching key players to positional requirements to optimize team formation.
van Arem et al., 2025 (68)	60,175 professional players; (Netherlands)	Male	Elite	86 multidimensional features including personal information, team quality, league level, contract details and transfer value, etc.	RF*, OLS, Lasso, DT, XGBoost, KNN	Regression/forecasting: future quality and transfer value	Python	Not Specified	Predicting players’ future development and value trajectories to identify those with growth potential for recruitment or contract decisions.
Yigit et al., 2020 (71)	5,316 outfield professional players (Football Manager + Transfermark); (Game)	Male	Elite	Player attributes from Football Manager, including technical, physical traits, age, league level, etc.	Lasso*, Ridge, RF, XGBoost	Regression: market value	R	MSE = 0.590	Predicting players’ real market value to support club transfer decisions and identify value-based talent.
									

ABPNN, adaptive back propagation neural network; ANN, artificial neural network; APSO, adaptive particle swarm optimization; AUC, area under the curve; BLS, broad learning system; CV, cross-validation; DFL, deutsche fußball liga; DNN, deep neural network; DT, decision tree; FMS, fundamental movement skills; GBC, gradient boosting classifier; GRFE, generalized recursive feature elimination; GRNN, graph recurrent neural network; KNN, K-nearest neighbours; LDA, linear discriminant analysis; LR, logistic regression or linear regression as reported in the original study; MLP, multilayer perceptron; MT-RR, multitask ridge regression; MT-SVR, multitask support vector regression; NB, naive bayes; NR, not reported; OLS, ordinary least squares; PCA, principal component analysis; PSO, particle swarm optimization; RDBLS, recurrent discriminative broad learning system; RF/RFC, random forest/random forest classifier; RFE, recursive feature elimination; RMSE, root mean squared error; SVM/SVR, support vector machine/support vector regression.

*Best-performing or selected model in the corresponding study.

All studies focused on male football players. Most samples involved athletes at the Elite level, whereas studies involving youth athletes generally examined Trained or Highly Trained players. Geographically, the included studies were predominantly conducted in Europe.

The included studies exhibited substantial variation in sample characteristics. Five studies used testing data from youth players (U8–U20), twelve studies employed professional football tracking and event datasets collected by third-party data providers, and three studies analyzed virtual player datasets derived from FIFA or Football Manager.

Traditional machine learning (ML) was the most frequently applied AI technique. Among all models, Random Forest (RF) or Random Forest Classifier (RFC) was used most often, appearing in 8 studies. This was followed by Lasso regression, which appeared in 5 studies. In addition, XGBoost, Ridge regression, and artificial neural networks (ANN) each appeared in several studies, with these models differing in their levels of interpretability. By contrast, deep learning (DL) and specialised or hybrid modelling approaches were less frequently applied. Unsupervised learning, dimensionality-reduction, or clustering methods were used in a small number of studies.

The input features used in AI models were multidimensional, including physical and fitness attributes (e.g., speed, jump performance, endurance, height and weight), technical–tactical indicators (event data, positional data, passing statistics, offensive and defensive actions), psychological factors, maturity-related measures (e.g., biological maturation, perceived competence), and career-related information (e.g., league level, match experience, injury history).

Software used across the studies was diverse, with Python, R, and MATLAB being the most common. Six studies did not report the software used.

Performance metrics also varied considerably. Classification-oriented studies commonly employed indicators such as accuracy, precision, recall, F1-score, AUC, or balanced accuracy, whereas regression or forecasting studies generally used error-, loss-, or correlation-based metrics such as MSE, RMSE, MAE, R^2^, or correlation coefficients. A small number of studies did not clearly report conventional model performance metrics.

Only a limited number of studies reported the availability of datasets or code, and most studies did not provide sufficient information to support reproducibility. In particular, only one study clearly provided a publicly accessible dataset, whereas most studies did not report code availability.

Overall, AI applications in football talent identification can be categorized into three core directions: (1) Developmental Potential Identification, focusing on predicting youth players’ future potential and likelihood of progression; (2) Performance & Role Identification, focusing on identifying high-level performance and key positional roles; (3) Player Value & Recruitment Identification, focusing on predicting market value, future player development, or supporting scouting and recruitment decisions. Collectively, existing research findings suggest that artificial intelligence has certain application potential in football talent identification.

## 4 Discussion

This systematic review found that AI has been widely applied across different stages of football talent identification, and that various application scenarios involve distinct data sources and choices of AI models. These differences not only reflect the current state of AI development but also reveal the inherent characteristics and practical needs of football talent identification systems.

From a macro perspective, talent identification is not a one-off or isolated event; rather, it is embedded within a dynamic talent development system (11). This system typically consists of talent detection (identifying potential athletes suitable for inclusion in talent development programs), identification (formally inviting athletes into these programs), and selection (evaluating athletes’ performance within the development process to determine whether they remain, progress, or exit the system) (38). These stages are supported by ongoing talent development and environmental facilitation, which together provide conditions that help athletes realize their potential (39, 40). Importantly, this process does not follow a single linear trajectory. For example, athletes who are initially deselected may continue participating in football and later be reconsidered in subsequent selection cycles, potentially entering higher-level development pathways (41). Therefore, talent identification should be regarded as a long-term, continuously updated process, one that can be increasingly supported and enhanced by advances in artificial intelligence.

Youth is generally regarded as a critical window for football talent identification, which is also reflected in the common practices of youth development systems worldwide (11). Today, practitioners and youth academies have shifted away from traditional talent identification approaches and have begun to evaluate and discover young prospects using multidimensional data (42). Compared with conventional statistical methods, current research is increasingly exploring the use of AI to optimize this process (24, 43, 44). Most of these studies employ machine learning methods. As a subset of artificial intelligence, machine learning has already been widely applied in the sports domain (29). Its advantages lie in its ability to analyze large datasets and continuously adjust and optimize model performance as new data become available (45). Specifically, Random Forest, XGBoost, and various regression models are the most frequently used techniques in youth talent identification. These models may be particularly suitable for structured, multidimensional youth datasets because they provide a practical balance between predictive flexibility, interpretability, computational feasibility, and robustness. Random Forest is capable of handling large sets of intercorrelated variables and performs well in classification tasks (46). XGBoost is likewise suitable for both classification and regression tasks. Regression-based models tend to demonstrate strong stability, particularly when applied to smaller sample sizes (47).

However, although these models demonstrate potential in youth talent identification, several challenges remain at the application level. Most machine learning algorithms are highly sensitive to the quality of input variables (48). In practice, the multidimensional data collected from youth players often contain inconsistencies in testing methods, evaluation standards, and operational procedures, all of which may affect model accuracy and stability (13). It is also important to note that, particularly during adolescence, factors such as biological maturation and relative age inherently introduce heterogeneity into player performance (49). This means that although machine learning and other AI methods are capable of capturing nonlinear relationships, if the training data already contain bias, the resulting talent identification models may unintentionally reinforce these biases rather than correct them.

Second, machine learning models rely on stable input distributions and sufficiently large sample sizes (28). However, in real youth academy environments, available samples are often limited, and data involving minors are subject to privacy constraints and institutional data management regulations (50). Consequently, limited sample size and uneven data distribution increase the risk of overfitting, reducing the model’s ability to generalize across different teams, age groups, or competition levels.

Moreover, most studies still rely on cross-sectional data for model development, using performance measures collected at a single time point to predict players’ developmental trajectories (43, 44). However, youth talent development is not a linear process; growth in technical, physical, and psychological domains often occurs in discontinuous or stage-like patterns, which cross-sectional models are unable to fully capture (32). The absence of longitudinal databases spanning multiple seasons or training centers further limits the ability of AI to accurately model player development trajectories, which represent a core determinant of long-term potential (11). In other words, these models tend to “predict players who currently fit the system,” but may not necessarily identify “players who could become outstanding in the future.” Because of these structural challenges, AI-although showing promising performance in youth talent identification-has not yet reached the point where it can replace traditional evaluation methods or comprehensively support decision-making in youth development systems.

Compared with youth talent identification based primarily on testing data, talent identification based on match performance relies on more complex tracking and event datasets (51). These data capture not only players’ technical-tactical actions but also spatiotemporal information such as position, speed, and spatial relationships (52). Given the continuous nature of football, its dynamic spatial structure, and its strong tactical dependencies, player performance cannot be adequately evaluated through single events or isolated technical indicators (53). Traditional linear models are often unable to capture the temporal and spatial patterns embedded in such data and therefore struggle to identify players who perform best within actual match contexts (54).

As a result, researchers have increasingly adopted methods beyond traditional machine learning, including neural network structures such as GRNN and ABPNN, dimensionality reduction techniques such as t-SNE and UMAP, and rule-based algorithms such as RIPPER (55). For example, Dick employed GRNN to quantify the value of players’ actions during matches (56), and Merlin used multiple models to compare passing patterns among players (51). The application of these methods reflects a growing trend in using AI to understand what players do in matches and how much their actions contribute to team performance, thereby providing a foundation for coaches and scouts to identify players who play key roles during competition. Action-valuation and possession-value frameworks—such as Valuing Actions by Estimating Probabilities (VAEP) (57), Expected Possession Value (EPV) (58), and related on-ball action value models, are also relevant to this broader trend, as they quantify the contribution of player actions or possessions in match contexts. However, these approaches primarily evaluate the immediate value of actions, possessions, or game states rather than player-level developmental potential, long-term performance trajectory, positional suitability, or recruitment value (59). Therefore, they were regarded as part of the wider football performance analytics literature rather than as core evidence for AI-based football talent identification (32).

Despite these strengths, the limitations are equally evident. One major limitation arises from the data itself. Tracking data are highly commercialized, lack public accessibility, and may be collected using different technologies and definitional standards across leagues, making it difficult for models to transfer and preventing their application across competitions (60).

At the same time, complex models impose higher methodological and computational demands, as their development requires extensive preliminary tuning, including feature selection, data normalization, temporal window segmentation, sample balancing, and parameter searching (61). The large volume and high-noise nature of tracking data further complicate this process (62). Modeling decisions made by different researchers—such as how to define an “action,” how to handle off-ball behavior, or how to segment tactical phases—can lead to differences between models that far exceed the performance differences attributable to the algorithms themselves, thereby reducing the comparability of research findings (63).

Finally, model interpretability remains a critical issue. Within contemporary academy and club environments, talent identification is increasingly recognized as a process that should integrate coaches’ subjective evaluations with objective measurements to support more comprehensive and effective decision-making (64, 65). In this context, data-driven approaches are not intended to replace coaches’ judgments, but rather to complement them as an additional source of information. However, if AI models provide only predictive outputs without clearly explaining the underlying rationale behind their decisions, their “black-box” nature makes it difficult for coaches and scouts to assess the validity of the results or integrate them with experiential knowledge. As a result, decision-makers may remain cautious or even reluctant to adopt such models, ultimately limiting their transparency and trustworthiness in practical applications (61, 66).

In the area of player value and recruitment identification, the studies included in this review employed the widest range of feature dimensions, including technical ability, physical characteristics, league level, contract information, and international reputation (67). Models such as Random Forest and XGBoost have become dominant in this domain (68). Compared with traditional linear modeling approaches, AI models offer distinct advantages in handling nonlinear relationships and integrating multidimensional information (69).

However, a potential issue in this line of research concerns the authenticity of the data sources. We observed that some existing studies rely on virtual datasets, such as those derived from FIFA or Football Manager (67). Although this approach may be feasible to some extent, from a methodological standpoint these datasets reflect the rules of a game world rather than the realities of actual football (70, 71). As a result, AI-generated predictions of “high-value players” may simply represent a fit to the virtual data system and may not necessarily translate into actionable decisions in real-world settings.

Overall, the application of AI in talent identification continues to face common limitations related to data quality, model selection, and interpretability (72). Specifically, limited external validation, insufficient reporting of model development and preprocessing procedures, and performance estimates derived from small or non-representative samples may introduce methodological bias into identification outcomes and lead to an overestimation of model performance (27). In addition, the lack of standardization in the reporting of model evaluation metrics, tuning procedures, and validation strategies across studies further undermines the quality and reproducibility of the evidence base (73–75). From a sports science perspective, particularly for practitioners directly involved in talent identification, highly complex indicator systems often lack practical utility. Instead, practitioners are primarily concerned with whether models are sufficiently accurate, whether they provide meaningful information to support the identification process, and whether they can be applied reliably within the dynamic and complex context of football (76).

Beyond these methodological limitations, structural biases in the application of AI to talent identification also warrant further attention, with gender bias being particularly prominent. This issue largely stems from the long-standing structural imbalance in research attention, data availability, and developmental resources within women’s football (77). From a talent identification perspective, the evaluation of female players is inherently more complex. Existing research suggests that menstrual cycle–related physiological factors may influence training status and match performance in female athletes (78), while a higher risk of anterior cruciate ligament (ACL) injuries may also have important implications for development continuity and career trajectories (79). These characteristics indicate that talent identification and developmental monitoring for female players cannot be directly based on evaluation frameworks derived from male samples. The direct application of such models to female talent identification may therefore result in biased evaluations of potential and, to some extent, reinforce existing inequalities. In addition to gender bias, the current body of research also shows a noticeable geographical concentration, with most studies conducted within European football contexts. This pattern may partly reflect the high availability of structured data from major European leagues and the dominant role of commercial data providers (80), but it may also limit the generalizability of findings to non-European environments.

In summary, although existing research suggests that AI has demonstrated promising potential in football talent identification, its application remains at an early stage of transition from methodological exploration to practical implementation. Accordingly, the findings of this study should be interpreted with caution and are more appropriately regarded as exploratory rather than directly applicable to practice. Future research should aim to improve data accessibility and sharing mechanisms, establish open benchmarks and standardized evaluation protocols, strengthen external validation using cross-club and cross-context datasets, promote the integration of multimodal data, and continue developing interpretable and trustworthy AI approaches. From a practical perspective, greater attention should also be paid to how AI outputs can be integrated into club decision-making processes, including human-AI collaboration in scouting, fairness across player populations, and the communication of model uncertainty to practitioners. With improvements in reproducibility, validation, interpretability, and practical governance, AI has the potential to evolve into a more reliable and practically valuable decision-support tool for youth development, player recruitment, and long-term performance evaluation.

## 5 Limitations and future directions

Several limitations should be considered when interpreting the findings of this review. First, the included studies were methodologically heterogeneous in terms of data sources, modelling aims, outcome definitions, and validation strategies, which limited direct comparison across studies and precluded quantitative synthesis. Second, only English-language studies were included, which may have introduced language bias and reduced the comprehensiveness of the evidence base. Third, grey literature and preprints were not systematically searched or included, which may have led to the omission of relevant and emerging evidence. Fourth, the participant profiles of the included studies were highly imbalanced. Most studies focused exclusively on male players, with very limited representation of female football populations. In addition, several studies were based on selective cohorts, single-team settings, or virtual datasets derived from football simulation platforms, which limits the representativeness and generalisability of the reported findings. Fifth, the risk-of-bias assessment showed that many studies lacked clearly reported external validation and provided insufficient detail regarding model development, preprocessing, and performance evaluation procedures. As a result, some reported model performances may be optimistic and should be interpreted with caution.

Future research should prioritise more representative and diverse football populations, particularly by increasing the inclusion of female players and by broadening the geographic and competitive scope of available datasets. Greater emphasis should also be placed on external validation, transparent reporting of preprocessing and tuning procedures, and the use of outcomes that more directly reflect real-world talent identification and recruitment decisions. In addition, future studies should strengthen data-sharing practices, improve reproducibility, and move beyond proof-of-concept designs toward more robust evaluation in real football environments.

## 6 Conclusion

This systematic review shows that artificial intelligence has been applied across several areas related to football talent identification. However, the present evidence base remains methodologically heterogeneous. Across the included studies, recurrent concerns were identified regarding sample representativeness, the use of virtual datasets, limited external validation, and insufficient analytical transparency. Accordingly, although the reviewed studies suggest that AI-based methods may support aspects of football talent identification, much of the current evidence should still be regarded as exploratory rather than practice-ready. Future progress in this field will depend on the development of more representative datasets, stronger validation strategies, clearer reporting standards, and more rigorous evaluation in real-world football settings. Under these conditions, AI may become a more reliable decision-support tool in football talent identification and player development.

## Data Availability

The raw data supporting the conclusions of this article will be made available by the authors, without undue reservation.
