# Supplementary material for: Artificial intelligence in football talent identification: a systematic review
Source: Front Sports Act Living. 2026 Aug 20;8:1770231. doi: 10.3389/fspor.2026.1770231 (PMC13538088; doi:10.3389/fspor.2026.1770231)
Supplement: Supplementary file 1 [file Table1.docx]

**Supplementary Table S1. Complete search strategies for each database.**

| **Database** | **Platform / search field** | **Exact search string** | **Filters / limits** | **Search date** | **Records retrieved** |
| --- | --- | --- | --- | --- | --- |
| Web of Science | Topic Search (TS) | TS=("artificial intelligence" OR "machine learning" OR "deep learning" OR "neural network") AND TS=("football" OR "soccer") AND TS=("talent" OR "talent identification" OR "scouting" OR "player selection" OR "player prediction" OR "transfer") | No publication-year restriction was applied. Eligibility restrictions on English-language, peer-reviewed original research articles were applied during screening. | June 15, 2025 | 249 |
| PubMed | MeSH terms and title/abstract | ("artificial intelligence"[Mesh] OR "machine learning"[Mesh] OR "neural networks, computer"[Mesh] OR "artificial intelligence"[tiab] OR "machine learning"[tiab] OR "deep learning"[tiab] OR "neural network"[tiab]) AND ("Soccer"[Mesh] OR football[tiab] OR soccer[tiab]) AND (talent[tiab] OR "talent identification"[tiab] OR scouting[tiab] OR "player selection"[tiab] OR "player prediction"[tiab] OR transfer[tiab]) | No publication-year restriction was applied. Eligibility restrictions on English-language, peer-reviewed original research articles were applied during screening. | June 15, 2025 | 109 |
| SportDiscus | Full text / TX | TX ("artificial intelligence" OR "machine learning" OR "deep learning" OR "neural network") AND TX ("football" OR "soccer") AND TX ("talent" OR "talent identification" OR "scouting" OR "player selection") | No publication-year restriction was applied. Eligibility restrictions on English-language, peer-reviewed original research articles were applied during screening. | June 15, 2025 | 155 |
| Scopus | Title, abstract, keywords | TITLE-ABS-KEY("artificial intelligence" OR "machine learning" OR "deep learning" OR "neural network") AND TITLE-ABS-KEY("football" OR "soccer") AND TITLE-ABS-KEY("talent" OR "talent identification" OR "scouting" OR "player selection" OR "player prediction" OR "transfer") | No publication-year restriction was applied. Eligibility restrictions on English-language, peer-reviewed original research articles were applied during screening. | June 15, 2025 | 93 |

**Note.** The searches were conducted on June 15, 2025. The numbers shown refer to records retrieved before duplicate removal. No publication-year restriction was applied at the database-search stage. Restrictions related to language, publication type, and empirical relevance were applied during the eligibility-screening process.
