# Supplementary material for: Artificial intelligence in football talent identification: a systematic review
Source: Front Sports Act Living. 2026 Aug 20;8:1770231. doi: 10.3389/fspor.2026.1770231 (PMC13538088; doi:10.3389/fspor.2026.1770231)
Supplement: Supplementary file 2 [file Table3.docx]

**Supplementary Table S3. PROBAST-informed domain-level risk-of-bias judgments and justifications.**

| **Study** | **Domain** | **Judgment** | **One-line justification** |
| --- | --- | --- | --- |
| Altmann et al., 2024 | Participants / data sources | Low | The study used a real-world youth academy cohort of 409 male U12–U19 players, although the sample was derived from a single academy context. |
|  | Predictors | Low | Predictors were clearly defined and included multidimensional testing and player-profile variables such as physical, technical, psychological, injury-related, relative-age, and positional indicators. |
|  | Outcomes | Low | The outcome was explicitly linked to player progression to higher age categories, which was directly relevant to developmental potential identification. |
|  | Analysis | Unclear | Internal model development and performance reporting were provided, but external validation using an independent academy, cohort, or season was not clearly reported. |
| Barron et al., 2018 | Participants / data sources | Low | The study used a real-world historical cohort of professional outfield players from English football, with a clearly defined player population. |
|  | Predictors | Low | Predictors were clearly derived from technical–tactical performance indicators and career-history variables. |
|  | Outcomes | Low | The outcome was explicitly related to progression to higher-level leagues, which was directly relevant to talent identification and recruitment potential. |
|  | Analysis | Unclear | The ANN-based modelling approach was reported, but model interpretability and external validation were limited or not clearly described. |
| Barron et al., 2020 | Participants / data sources | Low | The study used a real-world cohort of professional outfield players from English football, with a clearly defined player population. |
|  | Predictors | Low | Predictors were clearly derived from multidimensional technical, tactical, career, and international-competition variables. |
|  | Outcomes | Low | The outcome was explicitly related to identifying position-specific performance indicators and players with potential to progress to higher-level leagues. |
|  | Analysis | Unclear | Although position-specific ANN models were developed and performance was reported, model interpretability and external validation were limited or not clearly described. |
| Duncan et al., 2024 | Participants / data sources | Unclear | The study used a real-world youth grassroots sample, but the relatively small U8–U14 cohort limited representativeness and generalisability. |
|  | Predictors | Low | Predictors were clearly described and included anthropometric, maturation, fitness, fundamental movement skill, psychological, and coach-rating variables. |
|  | Outcomes | Unclear | The outcome was related to youth technical performance, but its direct alignment with long-term talent identification or developmental progression was less clearly established. |
|  | Analysis | High | The very high reported accuracy in a relatively small youth sample, combined with reliance on internal validation and no clearly reported external validation, raised substantial overfitting concerns. |
| Jauhiainen et al., 2019 | Participants / data sources | Low | The study used a relatively large real-world youth football sample with clearly defined U14 players. |
|  | Predictors | Low | Predictors were clearly described and included physical, technical, and self-assessment variables relevant to youth football development. |
|  | Outcomes | Unclear | The anomaly-detection target was related to identifying potential elite players, but the assumptions underlying the elite label and its developmental validity were not fully established. |
|  | Analysis | Unclear | The one-class SVM approach was appropriate for anomaly detection, but external validation and broader generalisability were not clearly reported. |
| Kelly et al., 2022 | Participants / data sources | Unclear | The study used a real-world academy sample, but the relatively small and selective cohort limited representativeness across broader youth football populations. |
|  | Predictors | Low | Predictors were clearly described and covered multidimensional technical–tactical, physical, psychological, and social variables relevant to youth development. |
|  | Outcomes | Unclear | The outcome was related to youth developmental potential, but the operational definition of talent progression was not as direct or standardized as formal selection or advancement outcomes. |
|  | Analysis | High | The exploratory modelling design, small sample size, limited emphasis on performance metrics, and lack of clearly reported external validation raised substantial risk of bias. |
| Sheng et al., 2020 | Participants / data sources | High | The study relied on videos from a single U20 men’s team, which substantially limited sample representativeness and real-world generalisability. |
|  | Predictors | Unclear | Predictors were derived from automated tracking and technical–tactical features, but feature construction and preprocessing procedures were not sufficiently transparent. |
|  | Outcomes | Unclear | The outcome involved automated scoring and ability assessment, but the operational link to standardized talent identification outcomes was not clearly established. |
|  | Analysis | High | The system-development focus, very high reported detection accuracy, limited validation transparency, and absence of clearly reported external validation raised substantial risk of bias. |
| Dick et al., 2021 | Participants / data sources | Low | The study used real-world tracking and event data from professional football matches, with a clearly defined elite competition context. |
|  | Predictors | Low | Predictors were derived from structured tracking and event data relevant to player actions and match performance. |
|  | Outcomes | Unclear | The outcome focused on player action quality, which is relevant to performance evaluation but only indirectly linked to talent identification or recruitment decisions. |
|  | Analysis | Unclear | The modelling approach and performance metrics were reported, but external validation and broader transferability across leagues or datasets were not clearly described. |
| García-Aliaga et al., 2021 | Participants / data sources | Low | The study used a large real-world dataset of professional European football players, providing a broad elite-level player sample. |
|  | Predictors | Low | Predictors were clearly derived from technical–tactical performance indicators across offensive, organisational, and defensive event categories. |
|  | Outcomes | Unclear | The outcome was based on player role or positional profiling, which is relevant to performance and scouting but only indirectly linked to formal talent identification. |
|  | Analysis | Unclear | Dimensionality-reduction and rule-based methods were reported, but validation against independent datasets or real recruitment decisions was not clearly described. |
| Khan et al., 2021 | Participants / data sources | High | The study was based on a single-player case using Lionel Messi’s career data, which severely limited representativeness and generalisability. |
|  | Predictors | Unclear | Predictors were simple match-level variables, but their construction and temporal relationship to real-world decision-making were not sufficiently clear. |
|  | Outcomes | High | The outcome focused on team dependence on one player, which is only weakly aligned with generalisable football talent identification. |
|  | Analysis | High | The single-player design, limited predictor set, low precision despite high accuracy, and absence of external validation raised substantial risk of bias. |
| Jamil et al., 2021 | Participants / data sources | Low | The study used a real-world sample of professional goalkeepers from European football, with a clearly defined elite and sub-elite comparison context. |
|  | Predictors | Low | Predictors were clearly derived from goalkeeper-specific technical performance indicators, including passing, distribution, catching, and shot-stopping variables. |
|  | Outcomes | Low | The outcome distinguished goalkeepers with UEFA Champions League experience from those without such experience, providing a clearly defined elite-performance classification. |
|  | Analysis | Unclear | Multiple machine learning models and performance metrics were reported, but external validation using an independent cohort or season was not clearly described. |
| Merlin et al., 2024 | Participants / data sources | Unclear | The study used real-world professional match data, but the dataset was limited to 2,522 passing events from only four matches, reducing representativeness. |
|  | Predictors | Low | Predictors were clearly derived from 2D positional data and technical–tactical variables related to pass difficulty and passing performance. |
|  | Outcomes | Unclear | The outcome focused on classifying pass difficulty and identifying superior passing players, which is relevant to performance evaluation but only indirectly linked to talent identification. |
|  | Analysis | Unclear | Multiple models and performance metrics were reported, but validation across independent matches, teams, or competitions was not clearly described. |
| Arndt & Brefeld, 2016 | Participants / data sources | Low | The study used real-world professional football data with a clearly defined player sample from the German football context. |
|  | Predictors | Low | Predictors were derived from match event data, player information, and team-level variables relevant to performance and recruitment evaluation. |
|  | Outcomes | Unclear | The outcome was related to future player performance or value, but its direct alignment with formal talent identification or real recruitment decisions was indirect. |
|  | Analysis | Unclear | Regression-based modelling and feature-selection approaches were reported, but external validation and broader transferability were not clearly described. |
| Behravan & Razavi, 2021 | Participants / data sources | High | The study relied on a FIFA 20 virtual/game-based dataset, which limited correspondence with real-world football populations and recruitment contexts. |
|  | Predictors | Low | Predictors were clearly defined within the FIFA dataset and included multidimensional player attributes related to technical, physical, defensive, offensive, and profile characteristics. |
|  | Outcomes | Low | The outcome was explicitly defined as player market-value estimation, which was directly relevant to recruitment and transfer decision-making. |
|  | Analysis | High | The use of a virtual dataset, lack of clearly reported external validation on real-world transfer data, and potential overfitting concerns raised substantial risk of bias. |
| Ćwiklinski et al., 2021 | Participants / data sources | Low | The study used a real-world professional football dataset with a relatively large sample of European players. |
|  | Predictors | Unclear | Predictors included player and team variables, but the construction and validity of some technical, psychological, and team-composite indicators were not fully transparent. |
|  | Outcomes | High | The transfer-success outcome was custom-defined and its alignment with real-world recruitment success or long-term player development was insufficiently validated. |
|  | Analysis | Unclear | Machine learning models and performance metrics were reported, but external validation and the robustness of the custom outcome definition were not clearly established. |
| Inan & Cavas, 2021 | Participants / data sources | Unclear | The study used a real-world professional sample, but it was restricted to a relatively small position-specific group of midfielders from one league and season. |
|  | Predictors | Low | Predictors were clearly defined match-performance variables, including minutes played, goals, expected goals, and assists. |
|  | Outcomes | Low | The outcome was explicitly defined as player market-value estimation, which was directly relevant to recruitment and transfer decision-making. |
|  | Analysis | High | The small position-specific sample, weak reported model performance, ANN opacity, and absence of clearly reported external validation raised substantial risk of bias. |
| Al-Asadi & Tasdemır, 2022 | Participants / data sources | High | The study relied on a FIFA 20 virtual/game-based dataset, which limited real-world representativeness and applicability to actual football recruitment contexts. |
|  | Predictors | Low | Predictors were clearly defined within the FIFA dataset and included player rating indicators such as age, height, potential, dribbling, shooting, position, and international reputation. |
|  | Outcomes | Low | The outcome was explicitly defined as player market-value prediction, which was directly relevant to recruitment and transfer decision-making. |
|  | Analysis | Unclear | Several machine learning models and performance metrics were reported, but validation on independent real-world football or transfer-market data was not clearly described. |
| Nouraie et al., 2023 | Participants / data sources | High | Although the dataset included a large number of professional players, the data source and sampling representativeness were insufficiently transparent for real-world recruitment generalisation. |
|  | Predictors | Unclear | Predictors included technical and physical attributes, but feature provenance, construction, and availability in real decision-making contexts were not fully transparent. |
|  | Outcomes | High | The outcome was based on player–position or team-formation matching, which was non-standard and only indirectly aligned with formal talent identification or recruitment success. |
|  | Analysis | High | The proof-of-concept DNN approach, limited interpretability, non-standard target outcome, and lack of clearly reported external validation raised substantial risk of bias. |
| van Arem et al., 2025 | Participants / data sources | Unclear | The study used a very large professional football dataset, but potential selection bias and the representativeness of the included players across broader football contexts were not fully clear. |
|  | Predictors | Low | Predictors were clearly defined and included multidimensional player, team, league, contract, and value-related variables. |
|  | Outcomes | Unclear | The outcome focused on future development and value trajectories, but these were partly model-derived and their alignment with real-world talent identification decisions was not fully established. |
|  | Analysis | Low | The study reported a comparatively transparent modelling framework using multiple benchmark models and a large dataset, reducing analytical risk relative to most included studies. |
| Yigit et al., 2020 | Participants / data sources | High | The study relied partly on Football Manager-derived virtual player attributes, which limited real-world representativeness despite linkage to market-value data. |
|  | Predictors | Low | Predictors were clearly defined and included technical, physical, age-related, and league-level attributes. |
|  | Outcomes | Low | The outcome was explicitly defined as real player market-value prediction, which was directly relevant to recruitment and transfer decision-making. |
|  | Analysis | Unclear | Multiple machine learning models and performance metrics were reported, but validation using independent real-world datasets and the robustness of virtual predictors were not clearly established. |

**Note.** The risk-of-bias framework was adapted from PROBAST and PROBAST+AI principles and tailored to the prediction-oriented artificial intelligence studies included in this review. Each study was assessed across four domains: participants/data sources, predictors, outcomes, and analysis. Judgments were classified as low, unclear, or high risk of bias. The justifications reflect information reported in the original studies; where relevant information was insufficiently described, this was considered in the judgment.
